# Functional Advantage of Central Pancreatectomy Over Distal Pancreatectomy for Benign or Low‐Grade Malignant Tumors: A Comparative Analysis Based on 75‐g Oral Glucose Tolerance Test

**DOI:** 10.1002/ags3.70139

**Published:** 2025-11-28

**Authors:** Dongha Lee, Keiko Kamei, Chihoko Nobori, Yuta Yoshida, Takaaki Murase, Atsushi Takebe, Yuki Okuda, Naru Babaya, Yoshihisa Hiromine, Ippei Matsumoto

**Affiliations:** ^1^ Department of Surgery, Faculty of Medicine Kindai University Minami‐ku, Sakai Osaka Japan; ^2^ Department of Endocrinology, Metabolism and Diabetes, Faculty of Medicine Kindai University Minami‐ku, Sakai Osaka Japan

**Keywords:** 75‐g oral glucose tolerance test, benign or low‐grade malignant tumor, central pancreatectomy, distal pancreatectomy, endocrine function

## Abstract

**Background:**

Central pancreatectomy (CP) is a function‐preserving procedure for benign or low‐grade malignant lesions in the pancreatic neck or proximal body. However, endocrine function after CP has not been objectively evaluated.

**Methods:**

We retrospectively analyzed 123 patients who underwent CP (*n* = 38) or distal pancreatectomy (DP; *n* = 85) for benign or low‐grade malignant tumors. Short‐ and long‐term outcomes were compared between groups. Among 37 patients (CP; *n* = 12, DP; *n* = 25) underwent a 75‐g oral glucose tolerance test (OGTT) before and 1 month after surgery to evaluate endocrine function.

**Results:**

CP was associated with a higher incidence of postoperative pancreatic fistula than DP (40% vs. 8%; *p* < 0.001). All CP patients achieved R0 resection with no perioperative mortality or tumor recurrence. The incidence of new‐onset diabetes mellitus was significantly lower after CP than after DP (14.8% vs. 50.9%, *p* = 0.002). In the DP group, the area under the curve (AUC) for blood glucose (BG) increased postoperatively (*p* < 0.001), whereas AUCs for immunoreactive insulin (IRI) and C‐peptide immunoreactivity (CPR) decreased (*p* = 0.010 and *p* = 0.022, respectively). Conversely, the CP group showed preserved endocrine function, with no significant postoperative changes in AUCs for BG (*p* = 0.351), IRI (*p* = 0.723), or CPR (*p* = 0.900).

**Conclusion:**

Compared with DP, which has acceptable short‐term outcomes, CP preserves pancreatic endocrine function. In patients with benign or low‐grade malignant pancreatic tumors with expected long‐term survival, CP should be considered over DP to preserve function.

## Graphical abstract

Central pancreatectomy (CP) better preserves postoperative pancreatic endocrine function than distal pancreatectomy for benign or low‐grade malignant tumors. The 75‐g oral glucose tolerance test demonstrated that CP maintained insulin secretion and glucose tolerance, highlighting its clinical advantage as a function‐preserving procedure.

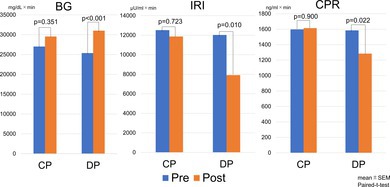

## 1 Introduction

The diagnosis of benign and low‐grade malignant pancreatic tumors, such as intraductal papillary mucinous neoplasms, serous cystic neoplasms, solid pseudopapillary neoplasms, and pancreatic neuroendocrine tumors, has become increasingly common owing to advances in diagnostic imaging. These tumors are frequently located in the pancreatic neck or proximal body, and selecting an optimal surgical approach is essential for balancing oncological curability with the preservation of both exocrine and endocrine functions.

Distal pancreatectomy (DP) is widely regarded as the standard surgical procedure for tumors in the neck or body of the pancreas [1, 2]. However, in patients with benign or low‐grade malignant tumors, DP may result in the unnecessary removal of substantial volumes of normal pancreatic parenchyma. The loss of pancreatic tissue increases the risk of postoperative exocrine and endocrine insufficiency. Indeed, previous studies have reported new‐onset diabetes mellitus (DM) rates of 30%–50% following DP [3, 4, 5, 6], which is particularly concerning in patients with benign or low‐grade tumors, who are expected to have long‐term postoperative survival. In contrast, central pancreatectomy (CP) is a parenchyma‐sparing procedure suitable for lesions confined to the pancreatic neck and proximal body. Although CP has been associated with a higher incidence of postoperative complications, including postoperative pancreatic fistula (POPF), several studies suggest that CP better preserves long‐term pancreatic function compared to DP [7, 8, 9, 10, 11]. However, most of these studies assessed endocrine outcomes solely using glycated hemoglobin (HbA1c). To the best of our knowledge, no study has compared CP and DP outcomes by objectively evaluating endocrine function using the 75‐g oral glucose tolerance test (OGTT) before and after surgery.

In this study, we retrospectively compared the short‐ and long‐term outcomes of CP and DP in patients with benign or low‐grade malignant pancreatic tumors. In particular, we assessed perioperative changes in glucose tolerance, insulin secretion, and C‐peptide levels using the OGTT. We aimed to clarify the functional advantages of CP over DP and discuss the clinical significance of CP as a function‐preserving surgical option.

## 2 Methods

### 2.1 Study Design and Patients

This retrospective, single‐center study included 123 consecutive patients who underwent either CP (*n* = 38) or DP (*n* = 85) for benign or low‐grade malignant pancreatic tumors between November 2010 and December 2023. The collected clinical data included age, sex, body mass index (BMI), preoperative DM, preoperative HbA1c levels, new‐onset DM, postoperative HbA1c levels, operative details, postoperative complications, pathological tumor size, and survival outcomes. Among them, a subgroup of 37 patients (CP; *n* = 12; DP; *n* = 25) underwent endocrine function testing using an OGTT before and 1 month after surgery, including measurement of blood glucose (BG), immunoreactive insulin (IRI), and C‐peptide immunoreactivity (CPR) levels.

This study was approved by the Ethics Committee of the Kindai University Faculty of Medicine (approval number: R06‐170) and conducted in accordance with the principles of the Declaration of Helsinki. The requirement for informed consent was waived because the retrospective nature of the study. Information regarding the study, including its purpose and the option to refuse participation, was disclosed on the institution's website in accordance with ethical guidelines.

### 2.2 Surgical Indications and Decision‐Making Process

The surgical procedure was determined at a multidisciplinary conference involving gastroenterologists, hepato‐biliary‐pancreatic surgeons, and oncologists. CP was considered for patients with benign or low‐grade malignant tumors adjudged curable by this procedure. In addition to this prerequisite, the following three criteria had to be fulfilled: absence of lymph node metastasis on preoperative imaging, ability to achieve negative surgical margins, and technical feasibility of reconstructing the gastrointestinal tract from the distal pancreatic stump.

### 2.3 Surgical Procedure of CP

All CP procedures were performed via open laparotomy. Laparotomy was performed through an upper midline incision. The lesser sac was opened by dividing the gastrocolic ligament, while preserving the gastroepiploic vessels. The superior mesenteric, portal, and splenic veins were carefully dissected free from the posterior aspect of the pancreas, paying attention to ligating multiple small side branches to the pancreas. The extent of the lymphadenectomy was determined based on the tumor's malignant potential. The lesion, located in the neck and body of the pancreas, was resected with sufficient margins in both the proximal and distal pancreatic parenchyma. Pancreatic transection was performed using a scalpel. After identifying the central pancreatic duct at the cut surface, direct double ligations were performed using a 4‐0 monofilament absorbable thread. The proximal pancreatic stump was wrapped with a strip of Vicryl mesh (woven type) and closed with a transpancreatic mattress suture using a 3‐0 monofilament polypropylene thread [12, 13]. Pancreaticodigestive reconstruction at the distal pancreatic stump was performed via pancreaticogastrostomy using the one‐layer invagination technique.

### 2.4 Surgical Procedure of DP

In DP, both open and laparoscopic approaches were used according to tumor location and size. For laparoscopic DP, pancreatic transection was performed using a reinforced triple‐row stapler with polyglycolic acid mesh (Endo GIA Reinforced Reload with Tri‐Staple Technology, Covidien, Tokyo, Japan), employing the slow‐firing method as previously described [14]. For open DP, the pancreatic stump was closed in the same manner as in CP—by direct double ligation of the main pancreatic duct, wrapping of the proximal stump with Vicryl mesh, and transpancreatic mattress sutures using 3–0 polypropylene [12, 13].

### 2.5 Postoperative Complications and Their Definitions

Postoperative complications were evaluated using the modified Clavien–Dindo classification system [15]. A complication classified as grade III or higher was considered clinically significant. POPF was assessed according to the International Study Group on Pancreatic Fistula (ISGPF) criteria [16]. Postoperative pancreatic hemorrhage (PPH) was evaluated according to the International Study Group of Pancreatic Surgery definition [17].

### 2.6 Endocrine Function Evaluation

New‐onset DM was defined based on the American Diabetes Association criteria, including either two or more fasting plasma glucose (FPG) levels ≥ 126 mg/dL, or a combination of FPG ≥ 126 mg/dL and HbA1c ≥ 6.5% [18]. Postoperative HbA1c levels were measured 6, 12, 24, and 36 months after surgery.

In a subgroup of 37 patients who underwent pancreatic function testing, endocrine function was further assessed by calculating the area under the curve (AUC) for BG, IRI, and CPR during the OGTT before and 1 month after surgery. The AUC values were compared between the preoperative and postoperative periods within each surgical group (CP and DP). Patients who underwent OGTT in this study were participants in the Kindai Prospective Study on Metabolism and Endocrinology after Pancreatectomy (KIP‐MEP study), a prospective observational cohort conducted at Kindai University to evaluate perioperative endocrine function [19]. The KIP‐MEP study recruited participants who met the following criteria: no preoperative diabetes, scheduled to undergo pancreatectomy, aged > 20 years, and provided written informed consent to participate.

### 2.7 Follow‐Up Management and Survival

Follow‐up data were collected until September 30, 2024. Routine surveillance was performed using computed tomography every 6–12 months postoperatively. If recurrence was suspected, additional magnetic resonance imaging and/or 18F‐fluorodeoxyglucose positron emission tomography was performed. Overall survival (OS) was defined as the time from surgery to death from any cause or the last follow‐up. Recurrence‐free survival (RFS) was calculated from the date of surgery to the date of recurrence or last follow‐up.

### 2.8 Statistical Analysis

All statistical analyses were performed using JMP software (version 16; SAS Institute, Japan). Patient characteristics and continuous variables were estimated as mean ± standard deviation (SD) or median (range), as appropriate. Categorical variables were determined as numbers and percentages. OS and RFS were estimated using the Kaplan–Meier method. Categorical variables were compared using Pearson's chi‐squared test. For patients who underwent the OGTT before and after surgery, paired *t*‐tests were used to compare the preoperative and postoperative AUC values for BG, IRI, and CPR within each surgical group (CP or DP). For graphical presentation of OGTT data, AUC values are expressed as mean ± standard error of the mean (SEM). A *p*‐value < 0.05 was considered statistically significant.

## 3 Results

### 3.1 Demographic and Clinical Characteristics of the Patients

The demographic and clinical characteristics of the patients are summarized in Table 1. A total of 123 patients were included in this study; 38 underwent CP and 85 underwent DP. No significant differences were noted between the CP and DP groups in terms of mean age, sex distribution, preoperative DM, or preoperative HbA1c levels. However, the CP group had a significantly higher BMI (23.7 ± 2.9 vs. 22.0 ± 3.6 kg/m^2^, *p* < 0.001) than the DP group did. The distribution of pathological diagnoses also significantly differed between the two groups (*p* = 0.015), with mucinous cystic neoplasms being more prevalent in the DP group.

**TABLE 1:** Demographic and clinical characteristics of the patients.

Values	CP (*n* = 38)	DP (*n* = 85)	p‐value
Age, years	63 ± 13	63 ± 13	0.889
Male patients	14 (37)	33 (39)	0.835
Preoperative BMI (kg/m^2^)	23.7 ± 2.9	22.0 ± 3.6	< 0.001
Preoperative DM	8 (21)	17 (20)	0.893
Preoperative HbA1c levels (%)	6.1 ± 0.8	6.0 ± 0.8	0.683
Pathology			0.015
IPMN/IPMC	9/4 (34)	28/7 (41)	
pNET	8 (21)	19 (22)	
MCN	1 (3)	18 (21)	
SCN	5 (13)	5 (6)	
High‐grade PanIN/pT1	4/1 (13)	4/0 (5)	
SPN	2 (5)	3 (4)	
Others	4 (11)	1 (1)	

*Note:* Values are means ± SD or *n* (%).

Abbreviations: BMI, body mass index; CP, central pancreatectomy; DM, diabetes mellitus; DP, distal pancreatectomy; HbA1c, glycated hemoglobin High‐grade; PanIN, high‐grade pancreatic intraepithelial neoplasia; IPMC, intraductal papillary mucinous carcinoma; IPMN, intraductal papillary mucinous neoplasm; MCN, mucinous cystic neoplasm; pNET, pancreatic neuroendocrine tumor; pT1, pathological T1 stage according to the American Joint Committee on Cancer/Union for International Cancer Control TNM classification, 7th edition, SCN, serous cystic neoplasm; SPN, solid pseudopapillary neoplasm.

### 3.2 Perioperative Outcomes of the Patients

The perioperative outcomes are summarized in Table 2. No significant differences were noted between the CP and DP groups in terms of the operative time or intraoperative blood loss. All CP procedures were performed via open laparotomy. In contrast, among patients who underwent DP, 49 (58%) underwent open surgery and 36 (42%) underwent laparoscopic surgery (*p* < 0.001). R0 resection was achieved in all patients in both groups (100%, *p* = 1.000). The incidence of surgical complications (Clavien–Dindo classification ≥ Grade IIIa) was significantly higher in the CP group than in the DP group (42% vs. 17%, *p* = 0.002). Clinically relevant POPF (ISGPF Grade ≥ B) occurred significantly more frequently in the CP group (40% vs. 8%, *p* < 0.001), and the postoperative hospital stay was significantly longer (16 vs. 11 days, *p* < 0.001) than in the DP group. However, no mortality was observed (0% in either group, *p* = 1.000).

**TABLE 2:** Intraoperative findings and short‐term results of the patients.

Values	CP (*n* = 38)	DP (*n* = 85)	*p*
Operative time, min	240 (133–362)	220 (75–427)	0.358
Intraoperative blood loss, mL	260 (5–1722)	300 (5–4398)	0.703
Intraoperative blood transfusion	1 (3)	10 (12)	0.101
Surgical approach, Open/Laparoscopic surgery	38/0	49/36	< 0.001
Texture of remnant pancreas, soft	37 (97)	81 (95)	0.590
Resection margin, negative	38 (100)	85 (100)	1.000
Clavien‐Dindo classification ≥ Grade IIIa	16 (42)	14 (17)	0.002
POPF≥Grade B	15 (40)	7 (8)	< 0.001
Postoperative pancreatic hemorrhage ≥ Grade B	2 (5)	3 (4)	0.653
Mortality	0 (0)	0 (0)	1.000
Postoperative hospital stay, days	16 (8–86)	11 (5–31)	< 0.001

*Note:* Values are presented as median (range), or number (%), as appropriate.

Abbreviations: CP, central pancreatectomy; DP, distal pancreatectomy; POPF, postoperative pancreatic fistula.

### 3.3 Survival Outcomes of the Patients

**FIGURE 1:**
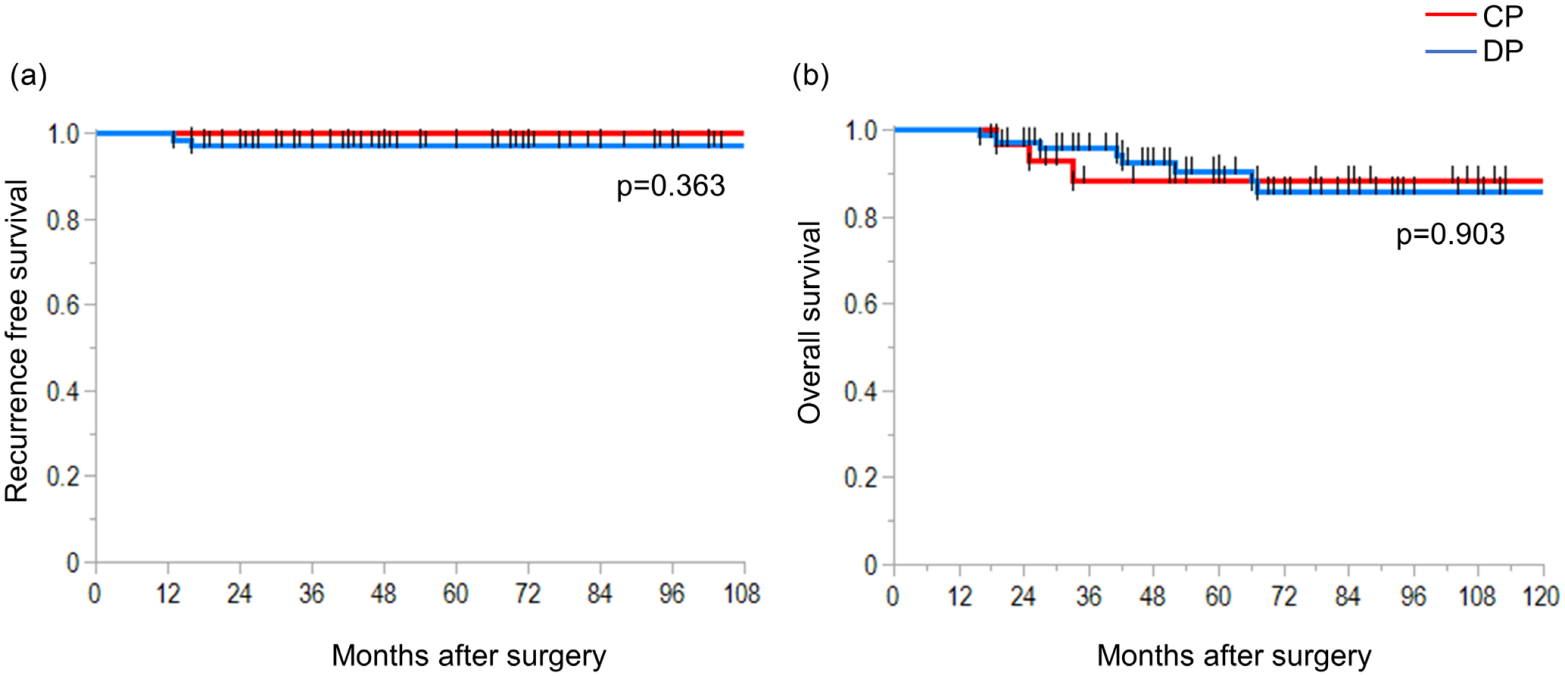
Kaplan–Meier curves comparing overall survival (OS) and recurrence‐free survival (RFS) between the central pancreatectomy (CP) and distal pancreatectomy (DP) groups. There were no significant differences between the two groups in OS (*p* = 0.903) or RFS (*p* = 0.363).

The median follow‐up period was 52 months (range, 1–154 months). No significant differences were noted in OS (*p* = 0.903) or RFS (*p* = 0.363) between the CP and DP groups (Figure 1). The 5‐year OS rates were 88% and 90% in the CP and DP groups, respectively, while the 10‐year OS rates were 88% and 86%, respectively. Tumor recurrence occurred in two patients in the DP group: one developed peritoneal dissemination 13 months after surgery for intraductal papillary mucinous carcinoma, and the other developed liver metastasis 16 months after surgery for a neuroendocrine tumor. No recurrence was observed in the CP group.

### 3.4 Evaluation of Endocrine Function Using OGTT

**FIGURE 2:**
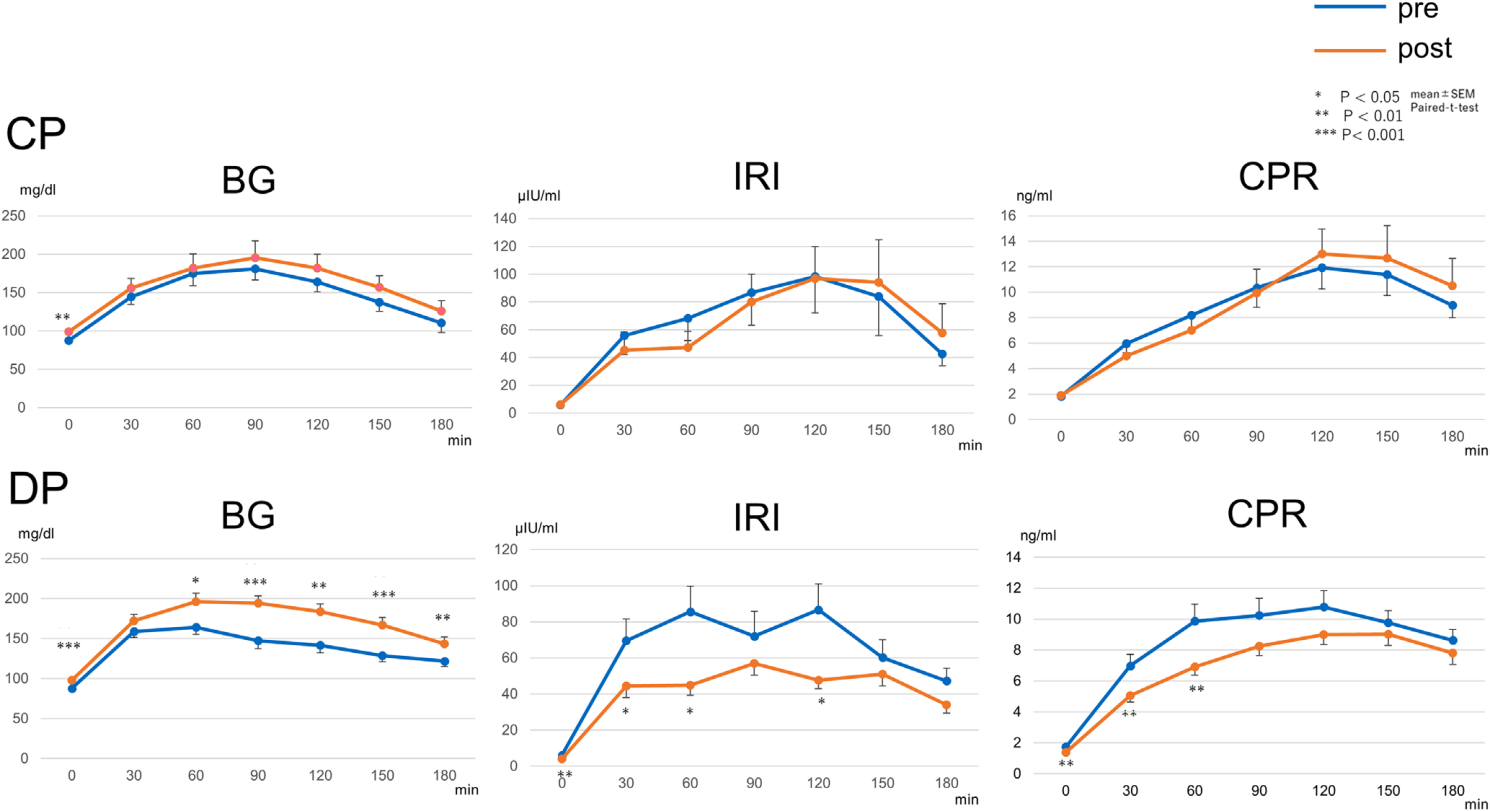
Changes in blood glucose (BG), immunoreactive insulin (IRI), and C‐peptide immunoreactivity (CPR) levels during the 75‐g oral glucose tolerance test (OGTT) before and 1 month after surgery in the central pancreatectomy (CP) and distal pancreatectomy (DP) groups. Blue lines indicate preoperative values, and orange lines indicate postoperative values. The CP group showed stable postoperative trends, while the DP group exhibited increased BG and reduced IRI and CPR levels postoperatively.

**FIGURE 3:**
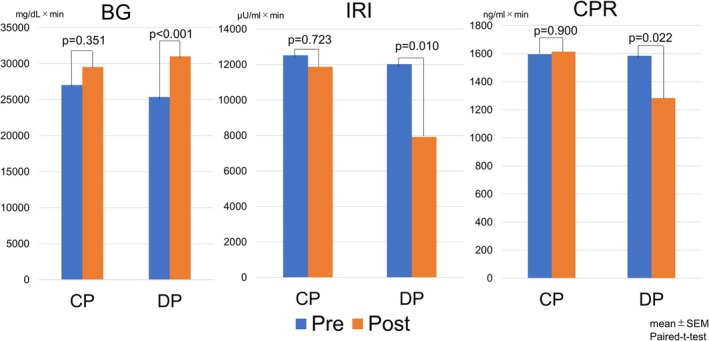
Comparison of area under the curve (AUC) values for blood glucose (BG), immunoreactive insulin (IRI), and C‐peptide immunoreactivity (CPR) levels during the 75‐g oral glucose tolerance test (OGTT) before and 1 month after surgery in the central pancreatectomy (CP) and distal pancreatectomy (DP) groups. Blue bars represent preoperative values, and orange bars represent postoperative values. In the CP group, there were no significant differences between preoperative and postoperative AUC values for BG (*p* = 0.351), IRI (*p* = 0.723), or CPR (*p* = 0.900), indicating preserved endocrine function. In contrast, the DP group showed a significant postoperative increase in BG AUC (*p* < 0.001), along with significant decreases in IRI AUC (*p* = 0.010) and CPR AUC (*p* = 0.022), suggesting a decline in glucose tolerance and insulin secretory capacity after surgery.

In a subgroup of 37 patients (CP, *n* = 12; DP, *n* = 25), endocrine function was evaluated using an OGTT before and 1 month after surgery. Baseline characteristics are summarized in Supplementary Table 1. There were no significant differences between the two groups in age, sex, preoperative HbA1c level, or pathological diagnosis, except that BMI was slightly higher in the CP group (23.9 ± 2.8 vs. 21.5 ± 3.3 kg/m^2^, *p* = 0.018). The changes in BG, IRI, and CPR over time are shown in Figure 2. The AUC was calculated for each parameter to quantitatively assess perioperative endocrine function. In the CP group, no significant differences were observed between the preoperative and postoperative AUC values for BG (*p* = 0.351), IRI (*p* = 0.723), or CPR (*p* = 0.900), indicating that endocrine function was preserved. In contrast, the DP group showed a significant postoperative increase in BG AUC (*p* < 0.001) and significant decreases in IRI AUC (*p* = 0.010) and CPR AUC (*p* = 0.022), suggesting a decline in glucose tolerance and insulin secretory capacity after surgery (Figure 3 and Supplemental Table 2).

### 3.5 Changes in HbA1c Levels and Incidence of New‐Onset DM

**FIGURE 4:**
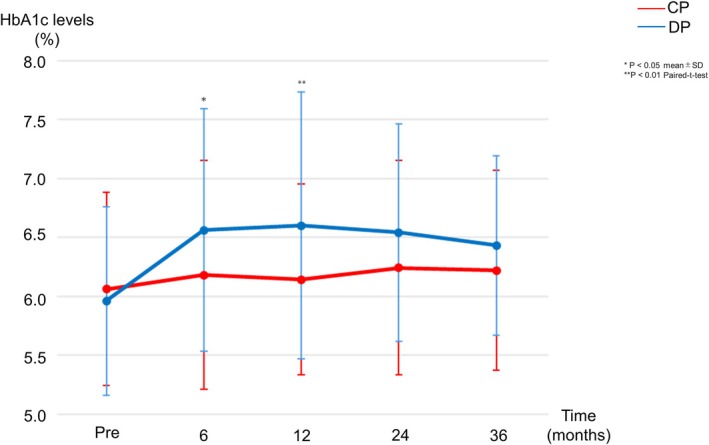
Glycated hemoglobin (HbA1c) levels were evaluated at baseline and during postoperative follow‐up at 6, 12, 24, and 36 months. The central pancreatectomy (CP) group demonstrated significantly better glycemic control at 6 and 12 months postoperatively compared to the distal pancreatectomy (DP) group.

Changes in HbA1c levels are summarized in Figure 4 and Supplemental Table 3. No significant difference was observed in the preoperative median HbA1c levels between the CP and DP groups (6.1% vs. 6.0%, *p* = 0.683). Postoperatively, the CP group exhibited significantly lower median HbA1c levels at 6 months (6.2% vs. 6.6%, *p* = 0.026) and 12 months (6.1% vs. 6.6%, *p* = 0.010) compared to the DP group. Among 27 patients who underwent CP and 55 who underwent DP without preoperative DM, in whom pre‐ and postoperative glucose and HbA1c measurements and follow‐up data were available, new‐onset DM developed in 14.8% (4/27) and 50.9% (28/55), respectively (*p* = 0.002). None of the CP patients required medical treatment for new‐onset DM, whereas 12 patients in the DP group (21.8%) required pharmacologic intervention, including insulin therapy in 7 and oral antidiabetic agents in 5 (*p* = 0.009). Among patients who developed new‐onset DM (CP; *n* = 4; DP; *n* = 28), there were no significant differences in age, sex, preoperative HbA1c level, or postoperative HbA1c changes between the two groups, except that BMI was slightly higher in the CP group (24.9 ± 2.5 vs. 21.7 ± 3.5 kg/m^2^, *p* = 0.046). All new‐onset DM cases in the CP group were mild and controllable with diet therapy alone (Supplementary Table 4). At 36 months after surgery, HbA1c levels did not differ significantly between the CP and DP groups, which was likely due to pharmacologic therapy, including insulin therapy, administered to several patients in the DP group, whereas none of the CP patients required medication.

## 4 Discussion

To the best of our knowledge, this is the first study to objectively demonstrate the functional advantage of CP over DP using perioperative OGTT‐based assessments, thereby providing a comprehensive understanding of the endocrine outcomes associated with these two procedures. This study compared CP and DP in patients with benign or low‐grade malignant pancreatic tumors, focusing on both short‐and long‐term surgical outcomes, as well as postoperative pancreatic endocrine function. Although CP was associated with a significantly higher incidence of POPF and overall complications than DP was, no perioperative mortality occurred, and R0 resection was achieved in all patients. Notably, despite its high morbidity, CP has clear advantages in preserving pancreatic endocrine function. This was supported not only by a significantly lower incidence of new‐onset DM but also by stable BG, IRI, and CPR responses on assessment using the OGTT. The OGTT enabled an objective and comprehensive evaluation of pancreatic endocrine function by measuring glucose tolerance and insulin secretory capacity, which cannot be assessed by HbA1c alone. While HbA1c reflects long‐term average glycemic control, the OGTT provides a real‐time assessment of glucose‐insulin homeostasis. In this study, analysis of the AUC for BG, IRI, and CPR revealed preserved endocrine function after CP but significant deterioration after DP, clearly demonstrating the superiority of OGTT over conventional HbA1c‐based evaluation. Previous studies have reported that new‐onset DM develops in approximately 30%–50% of patients after DP for benign or low‐grade malignant tumors [3, 4, 5, 6]. In our cohort, the incidence was even higher (50.9%), highlighting the substantial endocrine burden imposed by DP. Moreover, in our recent prospective study with a 3‐year follow‐up of 56 patients without diabetes who underwent DP, 33 patients (74.1%) developed new‐onset DM [20]. These findings further emphasize that in patients with benign or low‐grade malignant tumors, who are expected to have favorable long‐term prognoses, CP should be considered over DP for better preservation of pancreatic endocrine function, provided that appropriate patient selection is possible.

CP has been widely reported to preserve pancreatic endocrine function better than DP, particularly in patients with benign or low‐grade malignant tumors [9, 10, 11]. However, most previous studies have only assessed postoperative endocrine function using serum BG or HbA1c levels, which may not fully capture the physiological changes in insulin secretion or glucose regulation. In contrast, our study employed OGTT to objectively evaluate glucose metabolism, including IRI and CPR responses, both before and after surgery. We found that patients in the CP group maintained stable OGTT profiles postoperatively, whereas those in the DP group exhibited significant deterioration in glucose tolerance and insulin secretory capacity. In our study, all patients who underwent CP achieved R0 resection and remained recurrence‐free throughout the follow‐up period. These findings suggest that patients selected for CP are likely to have favorable long‐term outcomes. In such cases, preserving glycemic control and maintaining long‐term quality of life are especially important. Previous retrospective studies have reported new‐onset DM in approximately 5%–15% of patients following CP [9, 11, 21, 22, 23]. In the present study, the incidence of new‐onset DM was 14.8%. However, none of the patients required medical treatment during the follow‐up period. Therefore, for benign or low‐grade malignant tumors, CP is a clinically meaningful function‐preserving surgical option when implemented in appropriately selected patients.

Despite its functional advantages, CP has long been associated with a higher incidence of POPF compared to DP, owing to the presence of two transection margins and the need for pancreaticodigestive tract anastomosis [7, 9, 10, 11]. Over the past two decades (2000s to 2020s), several retrospective studies have reported clinically relevant POPF rates ranging from 30% to 40% following CP [9, 11, 21, 22, 23, 24, 25]. These findings are consistent with those of the present study, which demonstrated a 40% incidence of clinically relevant POPF in the CP group. However, in our cohort, no perioperative mortality occurred, and all patients achieved R0 resection and remained recurrence‐free during the follow‐up period. Although the relatively high POPF rate remains a challenge, the results suggest that the associated morbidity may be acceptable in appropriately selected patients with benign or low‐grade malignant tumors who are expected to have favorable long‐term prognoses. The incidence of POPF differed significantly among the three surgical approaches: 40% after CP, 12% after open DP, and 3% after laparoscopic DP (*p* < 0.001). These findings suggest that minimally invasive approaches may contribute to reducing the incidence of POPF compared with open procedures. In recent years, an increasing number of reports on robotic‐assisted CP have emerged [26, 27, 28]. Further studies are warranted to determine whether robotic‐assisted CP can help reduce the incidence of anastomosis‐related pancreatic fistula and shorten hospital stay through its minimally invasive advantages.

This study has several limitations. First, this was a retrospective analysis conducted at a single institution, and the sample size, particularly in the subgroup undergoing CP with OGTT, was relatively small. Second, the follow‐up duration, while sufficient to detect early postoperative outcomes, may not have been long enough to fully assess late endocrine dysfunction or tumor recurrence. Third, although the OGTT provides a quantitative assessment of endocrine function, it does not evaluate exocrine function, which also plays a crucial role in postoperative quality of life. Additionally, the decision to perform CP versus DP was made in a multidisciplinary setting, based on preoperative imaging and intraoperative judgment, which may have introduced selection bias. Future prospective multicenter studies with standardized assessment protocols and long‐term follow‐up are warranted to validate our findings and further refine the indications for CP in patients with benign or low‐grade malignant pancreatic tumors.

In conclusion, in preserving pancreatic endocrine function, CP provides a clear advantage over DP, which has acceptable short‐term outcomes. For benign or low‐grade malignant tumors with a favorable long‐term prognosis, CP should be considered as a function‐preserving surgical option.

## Author Contributions

**Dongha Lee:** conceptualization, writing – original draft. **Keiko Kamei:** data curation. **Chihoko Nobori:** data curation. **Yuta Yoshida:** data curation. **Takaaki Murase:** data curation. **Atsushi Takebe:** data curation. **Yuki Okuda:** data curation. **Naru Babaya:** conceptualization. **Yoshihisa Hiromine:** conceptualization. **Ippei Matsumoto:** conceptualization, writing – original draft.

## Funding

No funding was received for this study.

## Ethics Statement

This study was approved by the Ethics Committee of Kindai University Faculty of Medicine (Approval No. R06‐170). The protocol conformed to the provisions of the Declaration of Helsinki (as revised in Fortaleza, Brazil, October 2013). Informed consent was waived due to the retrospective nature of the study.

## Conflicts of Interest

The authors declare no conflicts of interest.

## Supporting information


**Supplemental Table 1** Baseline characteristics between the central pancreatectomy (CP) and distal pancreatectomy (DP) groups for the 37 cases that underwent a 75‐g oral glucose tolerance test (OGTT).

**Supplemental Table 2** Changes in the area under the curve (AUC) values for blood glucose (BG), immunoreactive insulin (IRI), and C‐peptide immunoreactivity (CPR) before and 1 month after surgery in 37 patients who underwent OGTT (CP, *n* = 12; DP, *n* = 25).

**Supplemental Table 3** Changes in serum glycated hemoglobin (HbA1c) levels before and after surgery in the central pancreatectomy (CP) and distal pancreatectomy (DP) groups.

**Supplemental Table 4** Comparison of clinical background and postoperative outcomes between the CP and DP groups among patients who developed new‐onset diabetes mellitus (DM).
