# Supplementary material for: Functional Advantage of Central Pancreatectomy Over Distal Pancreatectomy for Benign or Low‐Grade Malignant Tumors: A Comparative Analysis Based on 75‐g Oral Glucose Tolerance Test
Source: Ann Gastroenterol Surg. 2025 Nov 28;10(3):827–34. doi: 10.1002/ags3.70139 (PMC13178281; doi:10.1002/ags3.70139)
Supplement: Supplementary file 1 — Supplemental Table 1 Baseline characteristics between the central pancreatectomy (CP) and distal pancreatectomy (DP) groups for the 37 cases that underwent a 75‐g oral glucose tolerance test (OGTT). [file AGS3-10-827-s001.docx]

Supplemental Tables

Supplemental Table 1.

Baseline characteristics between the CP and DP group for the 37 cases that underwent OGTT

| Values | CP  (*n*=12) | DP  (*n*=25) | p-value |
| --- | --- | --- | --- |
| Age, years | 62±16 | 64±12 | 0.674 |
| Male patients | 3 (25) | 9 (36) | 0.503 |
| Preoperative BMI (kg/m^2^) | 23.9±2.8 | 21.5±3.3 | 0.018 |
| Preoperative HbA1c levels (%) | 5.8±0.1 | 5.7±0.1 | 0.414 |
| Pathology |  |  | 0.142 |
| IPMN / IPMC | 3/1 (33) | 5/2 (28) |  |
| pNET | 1 (8) | 8 (32) |  |
| MCN | 0 (0) | 5 (20) |  |
| SCN | 3 (25) | 1 (4) |  |
| High-grade PanIN / pT1 | 1/1 (17) | 3/0 (12) |  |
| SPN | 1 (8) | 0 (0) |  |
| Others | 1 (8) | 1 (4) |  |
| Operative time, min | 239 (133-297) | 235 (134-401) | 0.910 |
| Intraoperative blood loss, ml | 203 (5-1,772) | 30 (5-2,584) | 0.063 |
| Intraoperative blood transfusion | 0 (0) | 2 (8) | 0.314 |
| Surgical approach, Open / Laparoscopic surgery | 12/0 | 8/17 | <0.001 |
| Texture of remnant pancreas, soft | 12 (100) | 24 (96) | 0.482 |
| Resection margin, negative | 12 (100) | 25 (100) | 1.000 |
| Clavien-Dindo classification ≥ Grade Ⅲa | 4 (33) | 3 (12) | 0.121 |
| POPF ≥ Grade B | 4 (33) | 2 (8) | 0.050 |
| Postoperative pancreatic hemorrhage ≥ Grade B | 1 (8) | 1 (4) | 0.585 |
| Mortality | 0 (0) | 0 (0) | 1.000 |
| Postoperative hospital stay, days | 11 (8-80) | 8 (6-31) | 0.016 |

CP: central pancreatectomy, DP: distal pancreatectomy, OGTT: a 75-g oral glucose tolerance test

Values are means ± SD or n (%), median (range), or number (%), as appropriate.

BMI: body mass index, HbA1c: glycated hemoglobin

IPMN: intraductal papillary mucinous neoplasm, IPMC: intraductal papillary mucinous carcinoma,

pNET: pancreatic neuroendocrine tumor,

MCN: mucinous cystic neoplasm, SCN: serous cystic neoplasm,

High-grade PanIN: high-grade pancreatic intraepithelial neoplasia

pT1: pathological T1 stage according to the American Joint Committee on Cancer/Union for

International Cancer Control TNM classification, 7th edition

SPN: solid pseudopapillary neoplasm

POPF: postoperative pancreatic fistula
