# Supplementary material for: Functional Advantage of Central Pancreatectomy Over Distal Pancreatectomy for Benign or Low‐Grade Malignant Tumors: A Comparative Analysis Based on 75‐g Oral Glucose Tolerance Test
Source: Ann Gastroenterol Surg. 2025 Nov 28;10(3):827–34. doi: 10.1002/ags3.70139 (PMC13178281; doi:10.1002/ags3.70139)
Supplement: Supplementary file 2 — Supplemental Table 2 Changes in the area under the curve (AUC) values for blood glucose (BG), immunoreactive insulin (IRI), and C‐peptide immunoreactivity (CPR) before and 1 month after surgery in 37 patients who underwent OGTT (CP, n = 12; DP, n = 25). [file AGS3-10-827-s003.docx]

Supplemental Tables

Supplemental Table 2.

Changes in AUC values for BG, IRI, and CPR before and one month after surgery in 37 patients who underwent OGTT (CP; n = 12; DP; n = 25)

| Values | Group | Preoperative AUC values | Postoperative AUC values | p-value |
| --- | --- | --- | --- | --- |
| BG, mg/dL×min | CP | 27,018±1,906 | 29,548±2,273 | 0.351 |
|  | DP | 25,366±1,000 | 31,013±1,206 | <0.001 |
| IRI, µU/ml×min | CP | 12,520±2,975 | 11,871±2,477 | 0.723 |
|  | DP | 12,023±1,446 | 7,915±595 | 0.010 |
| CPR, ng/mL×min | CP | 1,597±208 | 1,615±228 | 0.900 |
|  | DP | 1,586±129 | 1,285±80 | 0.022 |

Data represent the subgroup of 37 patients who underwent OGTT (CP; n = 12; DP; n = 25).

AUC: area under the curve, BG: blood glucose, IRI: immunoreactive insulin, CPR: C-peptide immunoreactivity

OGTT: a 75-g oral glucose tolerance test, CP: central pancreatectomy, DP: distal pancreatectomy

Values are means ± SD
