# Supplementary material for: Functional Advantage of Central Pancreatectomy Over Distal Pancreatectomy for Benign or Low‐Grade Malignant Tumors: A Comparative Analysis Based on 75‐g Oral Glucose Tolerance Test
Source: Ann Gastroenterol Surg. 2025 Nov 28;10(3):827–34. doi: 10.1002/ags3.70139 (PMC13178281; doi:10.1002/ags3.70139)
Supplement: Supplementary file 3 — Supplemental Table 3 Changes in serum glycated hemoglobin (HbA1c) levels before and after surgery in the central pancreatectomy (CP) and distal pancreatectomy (DP) groups. [file AGS3-10-827-s004.docx]

Supplemental Tables

Supplemental Table 3.

Changes in serum HbA1c levels

| Values | CP  (*n*=38) | DP  (*n*=85) | p-value |
| --- | --- | --- | --- |
| Preoperative | 6.1±0.8 | 6.0±0.8 | 0.683 |
| 6 months after surgery | 6.2±1.0 | 6.6±1.0 | 0.026 |
| 12 months after surgery | 6.1±0.8 | 6.6±1.1 | 0.010 |
| 24 months after surgery | 6.2±0.9 | 6.5±0.9 | 0.079 |
| 36 months after surgery | 6.2±0.9 | 6.4±0.8 | 0.133 |

HbA1c: glycated hemoglobin

CP: central pancreatectomy, DP: distal pancreatectomy

HbA1c values are shown as mean ± SD (%)
