# Supplementary material for: Functional Advantage of Central Pancreatectomy Over Distal Pancreatectomy for Benign or Low‐Grade Malignant Tumors: A Comparative Analysis Based on 75‐g Oral Glucose Tolerance Test
Source: Ann Gastroenterol Surg. 2025 Nov 28;10(3):827–34. doi: 10.1002/ags3.70139 (PMC13178281; doi:10.1002/ags3.70139)
Supplement: Supplementary file 4 — Supplemental Table 4 Comparison of clinical background and postoperative outcomes between the CP and DP groups among patients who developed new‐onset diabetes mellitus (DM). [file AGS3-10-827-s002.docx]

Supplemental Tables

Supplemental Table 4.

Comparison of clinical background and postoperative outcomes between CP and DP groups among patients who developed new-onset DM

| Values | CP  (*n*=4) | DP  (*n*=28) | p-value |
| --- | --- | --- | --- |
| Age, years | 73±4 | 66±12 | 0.146 |
| Male patients | 2 (50) | 11 (39) | 0.683 |
| Preoperative BMI (kg/m^2^) | 24.9±2.5 | 21.7±3.5 | 0.046 |
| Preoperative HbA1c levels (%) | 6.1±0.8 | 6.0±0.8 | 0.683 |
| Pathology |  |  | 0.237 |
| IPMN / IPMC | 2/0 (50) | 9/2 (39) |  |
| pNET | 0 (0) | 3 (11) |  |
| MCN | 0 (0) | 8 (28) |  |
| SCN | 0 (0) | 2 (7) |  |
| High-grade PanIN / pT1 | 0/1 (25) | 3/0 (11) |  |
| SPN | 0 (0) | 0 (0) |  |
| Others | 1 (25) | 1 (4) |  |
| Operative time, min | 207 (133-296) | 244 (121-401) | 0.319 |
| Intraoperative blood loss, ml | 219 (171-775) | 256 (5-1,668) | 0.729 |
| Intraoperative blood transfusion | 0 (0) | 2 (7) | 0.581 |
| Surgical approach, Open / Laparoscopic surgery | 4/0 | 14/14 | 0.059 |
| Texture of remnant pancreas, soft | 3 (75) | 27 (96) | 0.098 |
| Resection margin, negative | 4 (100) | 28 (100) | 1.000 |
| Clavien-Dindo classification ≥ Grade Ⅲa | 1 (25) | 2 (7) | 0.252 |
| POPF ≥ Grade B | 1 (25) | 1 (4) | 0.098 |
| Postoperative pancreatic hemorrhage ≥ Grade B | 1 (25) | 0 (0) | 0.653 |
| Mortality | 0 (0) | 0 (0) | 1.000 |
| Postoperative hospital stay, days | 20 (15-58) | 9 (6-16) | 0.002 |
| Preoperative HbA1c levels | 6.2±0.2 | 5.8±0.1 | 0.039 |
| HbA1c levels 6 months after surgery | 6.5±0.4 | 6.7±0.2 | 0.647 |
| HbA1c levels 12 months after surgery | 6.4±0.7 | 6.9±0.3 | 0.743 |
| HbA1c levels 24 months after surgery | 6.7±0.4 | 6.8±1.1 | 0.563 |
| HbA1c levels 36 months after surgery | 6.4±0.0 | 6.7±0.2 | 0.636 |

CP: central pancreatectomy, DP: distal pancreatectomy, DM: diabetes mellitus

Values are means ± SD or n (%), median (range), or number (%), as appropriate.

BMI: body mass index, HbA1c: glycated hemoglobin

IPMN: intraductal papillary mucinous neoplasm, IPMC: intraductal papillary mucinous carcinoma,

pNET: pancreatic neuroendocrine tumor,

MCN: mucinous cystic neoplasm, SCN: serous cystic neoplasm,

High-grade PanIN: high-grade pancreatic intraepithelial neoplasia

pT1: pathological T1 stage according to the American Joint Committee on Cancer/Union for

International Cancer Control TNM classification, 7th edition

SPN: solid pseudopapillary neoplasm

POPF: postoperative pancreatic fistula
